# Prebiotic and Functional Fibers from Micro- and Macroalgae: Gut Microbiota Modulation, Health Benefits, and Food Applications

**DOI:** 10.3390/ijms262211082

**Published:** 2025-11-16

**Authors:** Nurdeniz Deniz, Sümeyye Sarıtaş, Mikhael Bechelany, Sercan Karav

**Affiliations:** 1 Department of Molecular Biology and Genetics, Çanakkale Onsekiz Mart University, Çanakkale 17000, Türkiye; 2 Institute Européen des Membranes (IEM), Unité Mixte de Recherche (UMR) 5635, University Montpellier, ENSCM, CNRS, F-34095 Montpellier, France

**Keywords:** algal polysaccharides, prebiotics, gut microbiota, health benefits, food application

## Abstract

Micro- and macro-algae are natural resources that attract attention in terms of their prebiotic potential and functional food applications due to their rich polysaccharide diversity. In this review, the regulatory effects of dietary fibers and polysaccharides from algae on gut microbiota, their health benefits and their potential functions in foods are discussed in detail. Compounds such as fucoidan, laminarin, alginate, porphyran, agar, carrageenan and exopolysaccharides are examined for their interactions with the microbiota and how they support digestive health, immunity and metabolic balance through the production of short chain fatty acids. In contrast to earlier reviews, this paper offers a comprehensive comparison between sulfated and non-sulfated algal polysaccharides, incorporates updated insights on their regulatory status and safety, and highlights emerging direction for developing next-generation prebiotic formulation. The review also examines their applications in functional foods, nutraceutical effects and protective roles, and includes preclinical and clinical studies. However, some limitations such as safety of consumption, risk of heavy metal accumulation, bioavailability issues and regulatory restrictions are also addressed. New nutritional approaches, next generation prebiotic formulations and biotechnological studies are included. This review aims to comprehensively highlight the versatile potential of algal polysaccharides as functional fibers and prebiotics. While numerous studies have examined algal polysaccharides, their heterogeneous structures and safety. This review emphasized these critical gaps and proposed a rational evaluation framework for future research and functional food development.

## 1. Introduction

There is an increasing interest in specific components that positively affect gut health, such as prebiotics and functional dietary fibers, with the realization of the relevance of gut health and microbiota on overall well-being [1,2]. Fibers encompass both ‘dietary fibers’ naturally found in foods and isolated fibers with proven positive physiological effects, known as ‘functional fibers’ [3]. Dietary fibers are complex carbohydrate polymers naturally found in plant-derived food products that are not digested or absorbed in the small intestine; they can pass into the large bowel and be fermented by the gut microbiota [4]. They are divided into four classifications: non-starch polysaccharides (NSP), resistant starch (RS), resistant oligosaccharides (RO), and lignin, according to EFSA [5]. To date, dietary fibers have been indicated to regulate bowel function, reduce blood glucose concentration, and lower blood cholesterol [6]. Therefore, they may reduce the risk of chronic diseases. Daily dietary fiber intake by the host is essential for a healthy and balanced gut microbiota. However, not every dietary fiber is a prebiotic [4]. Prebiotics can be defined as dietary fibers that are the substrate for beneficial bacteria in the digestive tract [7]. Each of the prebiotic substances has unique health benefits that can resist digestion in the upper intestinal tract [8]. They are fermented by the colonic microbiota and maintain gut health and microbiota balance by increasing short-chain fatty acid (SCFA) production and decreasing gut pH [9]. Nowadays, functional food products have become popular not only for their nutritional value but also for their positive impacts on health [10]. The growing interest in prebiotics has led to the increased significance of functional and supplementary food products that contain prebiotics, which offer numerous health benefits in the sector [11]. With this rising popularity, the search for sustainable and underutilized resources has emerged in the sector. Marine algae represent a promising resource for meeting these demands [12].

Algae are considered an environmentally friendly and sustainable resource containing many bioactive components. They are either eukaryotic or prokaryotic photosynthetic organisms that can live in both freshwater and saltwater. They range from micro-sized to enormous seaweeds [13]. Although only eukaryotic photosynthetic organisms are defined as algae, historically blue-green algae (cyanobacteria) were also defined as algae due to their similarity to algae and are still considered in this category today [14]. Many micro- and macroalgae species are well suited for human consumption [13]. Numerous nutraceuticals impact of algae has been reported to date. Since their biomass contains many biologically valuable compounds, including polysaccharides, proteins, fatty acids, lipids, and vitamins, they are used in many fields, such as the functional food and feed industry, pharmaceuticals, cosmetics, and biofuel production [15]. They demonstrated diverse bioactivities including antioxidant, anti-inflammatory, cardioprotective, prebiotic, and metabolic regulatory effects, as evidenced by in vitro and in vivo studies [16].

Prebiotics and functional fibers from micro- and macroalgae are considered the most remarkable natural and sustainable functional food production resources in this field due to their rich soluble polysaccharide content [16]. In addition, they contain many elements, such as pigments, phenolics, peptides, and high protein content, which exhibit bioactive properties [13]. Micro- and macroalgae are considered economically and eco-friendly resources due to their high biomass yields, rapid growth rates, and their ability to be cultivated in low-value media, such as wastewater [15]. Numerous researchers focus on the health effects of marine algae due to their rich and complex polysaccharide content [17,18]. Certain polysaccharides isolated from edible algae species exhibit prebiotic activity, increasing SCFA production, modulating gut microbiota, and facilitating blood sugar regulation by inhibiting certain enzymes that are involved in carbohydrate metabolism [19]. Additionally, seaweed polysaccharides have the potential to weight metabolism modulating [20], antioxidant [21], anti-inflammatory [22], anticancer, and immune response enhancing activities [23].

The widespread use of prebiotics in functional foods has prompted the search for many new sources. One of these resources is micro- and macroalgae, which are natural, sustainable, and economic [24]. The rich polysaccharide content of seaweeds can exhibit many bioactive properties and is of interest in the functional food industry [25]. These substances may exhibit prebiotic characteristics. They offer numerous health impacts, such as maintaining gut health by modulating gut microbiota, enhancing immune response, and reducing the risk of certain chronic diseases including diabetes, obesity, and cardiovascular disease [12,26]. However, research on prebiotic activities of algal polysaccharide (APS) is not sufficient and comprehensive [24,25].

Recent studies have demonstrated diverse applications of APSs. They are utilized in functional foods as thickeners, stabilizers, gelling agents, and emulsifiers [15]. Also, they contribute to prebiotic activity by modulating gut microbiota [16]. Additionally, APSs show potential nutraceutical and therapeutic formulations due to their immunomodulatory, antioxidant, and metabolic health promoting effects [15]. Although there are numerous studies on prebiotic and functional fibers from algae, the existing data in this field mostly focus on a single species or compound group, and comparative analyses remain limited. This review distinguishes itself from previous studies by comprehensively addressing the structural and functional differences in APSs. Furthermore, current safety assessment regulatory frameworks, and future biotechnological approaches are examined together providing a comprehensive evaluation of the potential of APSs in novel prebiotic formulations.

This review focuses on the prebiotic potential of polysaccharides derived from micro- and macroalgae. Particular emphasis is placed on their contribution to balancing the gut microbiota, the associated health benefits, and their current and future uses in the food and medical industry. As they are derived from natural, unexploited, and renewable resources, they can also play an important role in sustainable nutrition approaches.

## 2. Algal Polysaccharides

**Figure 1:**
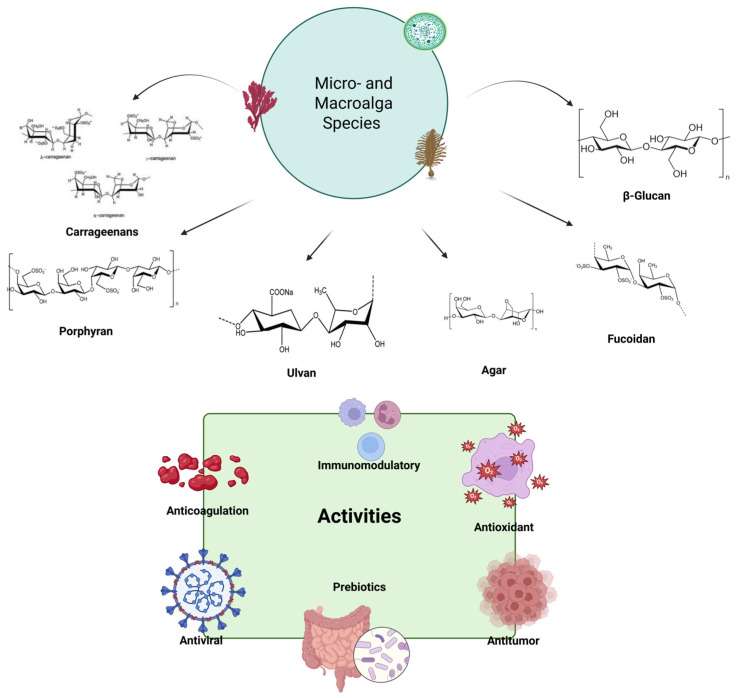
Algal polysaccharides and their bioactivities. This figure illustrates the main types of polysaccharides derive from micro- and macroalgae, including fucoidan, porphyran, carrageenans, agar, β-glucan and ulvan, and summarizes their key biological effects such as modulation of gut microbiota, enhancement of immune response, antioxidant activity, and support of metabolic health.

Algae contain rich bioactive compounds including proteins, lipids, minerals, vitamins, pigments, fatty acids, and polyphenols. They are also rich in valuable polysaccharide content [27]. Total polysaccharide contents vary from species to species. They range between 35% and 62% on average [25]. Both micro- and macroalgae serve as excellent, sustainable, biocompatible, and renewable sources for the extraction of polysaccharides (Figure 1). They can exhibit bioactive properties and be used in industries such as food and biomedicine [28]. Polysaccharides in algae can be found in the cell wall, intracellularly or in extracellular secretions [28]. APS is made of sugars like glucose, galactose, xylose, fucose, mannose, and arabinose. These sugars connect through glycosidic bonds. They may be sulfated, including ulvan, fucoidan, and carrageenan, or non-sulfated, such as laminarin and alginate. Agar can consist of both non-sulfated agarose and sulfated agaropectin [29].

The polysaccharides and fibers from seaweeds account for 33 to 62% of their dry weight, and they exhibit prebiotic activity in the gastrointestinal tract due to their resistance to human digestive enzymes [30]. They also demonstrate beneficial effects against a range of metabolic and chronic diseases, including diabetes, obesity, cancer, neurodegenerative diseases, liver disease, cardiovascular disease, and musculoskeletal disease [31]. Isolated polysaccharides derived from algae can vary based on the type of algae, e.g., those from macroalgae include alginate, laminarin, ulvan, fucoidan, carrageenan, and those from microalgae and cyanobacteria include, β-glucan, cellulose, pectin, agaropectin, chitin, and lignin [29]. APSs are also known as glycans [32]. Sulfated glycans, including carrageenan, ulvan, porphyran, fucoidan, and sulfated exopolysaccharides, are thought to be carbohydrates that regulate and influence the intestinal microbiota [33]. However, some studies have indicated that carrageenans have a negative effect on intestinal health [34,35].

### 2.1. Sulfated Polysaccharides

Sulfated APSs are complex macromolecules characterized by the presence of sulfate ester group covalently linked to their sugar residues. They exert many biological activities including antioxidant, antiviral, and anticoagulant effects that are largely influenced by their degree of sulfation. Sulfated APSs can be classified as fucoidan, carrageenan, ulvan, porphyran, and agar. Structural sulfation causes marked differences in functional activity by altering certain properties including viscosity and solubility. Sulfation significantly increases their biological activities, particularly their antioxidant and immunomodulatory effects [33,36].

#### 2.1.1. Fucoidan

Fucoidan is a sulfated polysaccharide mainly found in the cell walls of brown algae [37]. Its backbone is formed by α-fucopyranose units, most of which are sulfated fucose residues. They may also contain more than one sugar monomer such as galactose, mannose, glucose, and uronic acids. In this respect, the structure of fucoidan varies depending on the algal source. However, all structures exhibit similar properties [38]. It can constitute 25–30% of the dry weight of algae [39]

To date, numerous health benefits of fucoidan have been reported. These activities include antioxidants, antiviral, antitumor, anti-inflammatory, antibacterial, anticoagulant, antithrombotic, antifibrotic, and immunomodulatory effects. It has been previously tested as a natural source to manage bacteria that act as pathogens in the teeth and stomach, as well as the herpes virus that acts as a pathogen in the vagina [40]. It also has positive effects on various skin diseases. It has been found in the literature to have a positive effect on certain problems such as atopic dermatitis, skin carcinogenesis, and skin aging [41].

Beyond these, fucoidan has been studied for natural cancer treatment. Hoang et al. (2022) [42] reported to be effective against lung, colon, and breast cancer cells. In another article mentioned that fucoidan derived from *Fucus vesiculosus* and *Undaria pinnatifida* inhibited the growth of prostate and liver cancer cells [42]. Also, it showed suppressive effects on cell lines associated with colon cancer [43]. Additionally, fucoidan when used in the right dose, can act as a marine-derived prebiotic that promotes probiotic growth and inhibits the formation of harmful bacteria [40]. It is a promising polysaccharide with a wide range of potential uses in functional food, biomedical, and therapeutic applications due to its species-specific structural complexity and numerous bioactivities.

#### 2.1.2. Carrageenan

Carrageenans are linear, sulfated polysaccharides usually found in red algae such as *Kappaphycus alvarezii*, *Eucheuma denticulatum*, *Chondrus crispus*, and *Sarcothalia crispata* [44]. They consist of three groups: Kappa (κ), Iota (ι), and Lambda (λ), and each group has different characteristics. They can carry one, two or three sulfate groups. Their structure consists of D-galactose units [45]

Carrageenan have been applied in the food industry for decades for their water retention, thickening, gelling, and stabilizing effects. With the advancing food technologies, carrageenans are used in edible films and coatings, microencapsulation, 3D/4D printing technologies. Due to these properties, carrageenans are utilized in many food products, including meat, dairy, seafood, and bakery products [46]. They have numerous important biological activities, such as antiviral, anticoagulant, anticancer, and immunomodulatory. The degree and pattern of sulfation primarily influence their activity [34]. Carrageenans are known to be effective against many viruses, including coronaviruses. Also, they have potential antiviral effects against non-coronaviruses, among them influenza virus, human papillomavirus, rabies virus, Junín virus, Takibi virus, African swine fever virus, bovine herpesvirus, Suid herpesvirus and rhinovirus [47].

In contrast to these effects, some studies suggest that carrageenan causes inflammation. Although lambda carrageenan is mostly used to study the processes involved in inflammation, iota carrageenan is also used [34]. Additionally, carrageenans do not have any supportive effects on intestinal health. One study analyzed the effects of carrageenans on intestinal regulation and colitis [35]. The results showed that carrageenans did not significantly improve the symptoms of colitis and reduced SCFA production. Furthermore, carrageenan increased the abundance of *Ruminococcus gnavus* bacteria. The increase in this bacterium aggravated the symptoms of colitis. Another study reported that in addition to reducing SCFA production, kappa carrageenan also thinned the intestinal mucosa and weakened the intestinal barrier [17]. This exacerbated inflammation and tissue damage by increasing the action of pathogens such as *Citrobacter rodentium*.

Carrageenans have been used in the food industry for many years due to their thickening, gelling, and water retention effects. Also, they promise potential in the pharmaceutical industry [46]. Carrageenans, also known for their antiviral properties, show broad-spectrum activity against both enveloped and some non-enveloped viruses [48].

Carrageenan have shown clear pro-inflammatory effects in animal models. In a small number of studies in humans, they have also been associated with increased IBD symptoms and elevated inflammatory markers [49]. However, pro-inflammatory effects of carrageenans vary depending on type of carrageenan used, its concentration, and duration of exposure. High molecular weight carrageenan (200,000–800,000 Da) used in food is generally considered safe. But there is evidence that low molecular carrageenan triggers inflammation at higher concentration, while carrageenan form used in foods trigger inflammation at lower doses [49]. For example, a study conducted on patients with ulcerative colitis (UC) found data suggesting that carrageenan intake causes inflammation and increased disease activity [50]. The carrageenans, were defined as food-grade carrageenan, consisting of a mixture of kappa, lambda and iota types. For these reasons. The types of carragenaan and the dose administered are crititical in evaluating the inflammatory effect [49].

In light of this information, carrageenans are promising antivirals but may have a negative impact on gut health by causing inflammation.

#### 2.1.3. Ulvan

Ulvan is a cell wall polysaccharide that is mainly derived from green algae. It is mainly extracted from the genus *Ulva* [51]. The structure of ulvan, consisting of repeating disaccharide units, contains sulfated rhamnose, glucuronic acid, iduronic acid, and xylose. It constitutes 9 to 36% of the dry weight of the *Ulva*. For example, *Ulva* provides unique health impacts due to its high ulvan content. It can reduce the risk of certain chronic diseases by regulating intestinal health [52]. Such effects are specific to *Ulva* and are not directly related to ulvan. It is a bioactive polysaccharide that has attracted attention for its potential applications in functional foods, exhibiting antioxidant, antiviral, anticoagulant, and anti-inflammatory properties. According to Liu et al. (2022), ulvan may play a role in regulating genes associated with aging and contribute to the maintenance of gut microbiota balance [53]. Similarly, Pradhan et al. (2023) emphasized that ulvan could be considered a potential anticancer agent due to its immunomodulatory effects [54].

In addition, ulvan represents a natural and sustainable source of biomaterial, with the ability to be processed into gels, nanomaterials, fibers, and films [55]. It extracted from *Ulva rigida* has been combined with biocompatible polymers to produce nanofiber biocomposites, which show promise for biomedical applications such as tissue engineering, drug delivery, and wound healing [56]. However, further studies are required to validate the efficacy and safety of these biomedical uses.

#### 2.1.4. Porphyran

Porphyran is a sulfated polysaccharide extracted from red algae of the genus Porphyra, where it constitutes approximately 11–21% of the dry biomass [57]. Structurally, it is characterized by alternating residues of galactose and 3,6-anhydrogalactose, together with galactose-6-sulfate and 6-O-methyl-galactose units [25]. A growing body of evidence indicates that porphyran exerts diverse biological activities, including antihyperlipidemic, antioxidant, immunomodulatory, antitumor, antidiarrheal, and prebiotic effects. Qiu et al. (2020) highlighted its broad nutraceutical potential, suggesting that porphyran could serve as a valuable natural ingredient in functional foods [58]. In vitro studies further demonstrated its capacity to scavenge reactive oxygen species such as superoxide anion (O_2_^−^) and hydroxyl radical (OH^•^), thereby confirming its strong antioxidant properties [57]. Anti-inflammatory actions have also been reported, particularly through the inhibition of nitric oxide production.

Beyond these systemic effects, porphyran contributes to intestinal health by enhancing epithelial cell migration and proliferation, which supports wound healing processes [58]. Xu et al. (2019) reported that porphyran can increase SCFA production in the gut, thereby promoting a favorable microbial environment [59]. Consistently, porphyran supplementation has been shown to stimulate the growth of beneficial probiotic bacteria while suppressing harmful pathogens. Taken together, these findings underscore porphyran’s multifunctionality and reinforce its promise as a bioactive polysaccharide with applications in functional food development and pharmaceutical formulations.

#### 2.1.5. Agar

Agar is a complex hydrophilic colloid primarily composed of two fractions, agarose and agaropectin, and is generally extracted from red algae. Agarose has been widely studied because of its defined structure and broad applications [29]. Agaropectin, on the other hand, is less understood since its composition is highly complex and harder to apply in practice [60]. Unlike most sulfated polysaccharides, agar is not inherently rich in sulfate groups, although sulfate substituents may still be present.

Its primary commercial use is in the food industry. Agar functions as a stabilizer, hydrocolloid, emulsifier, flocculant, gelling agent, humectant, and thickener [61]. Yet, it also faces challenges. Its large molecular size, limited solubility, and strong hydrocolloidal nature restrict wider applications [60]. In addition to its frequent use in the food industry, agar has many important benefits, just like other APSs [29]. Oligosaccharides obtained from agarose are indigestible by enzymes in the upper part of the human digestive system. A study found that AOS exhibited prebiotic properties by promoting the growth of beneficial bacteria in the colon [62]. In addition, it has shown better pathogen inhibition than galacto-oligosaccharides, which are commercial prebiotics.

Agar oligosaccharides have also been reported to possess various biological activities including antioxidant, anticancer, anti-inflammatory, anti-melanogenesis, immunomodulatory, antilipidemic, and hypocholesterolemic activities [62].

### 2.2. Non-Sulfated Polysaccharides

Non-sulfated APSs lack sulfate group in their molecular structure and are mainly composed of neutral sugar monomers and uronic acids. These compounds, such as laminarin and alginate are found particularly in brown algae. They primarily serve structural or storage functions. Non-sulfated APSs also exhibit different physicochemibal properties than their sulfated counterparts [28,63].

#### 2.2.1. β-Glucan

β-glucans are a large class of dietary polysaccharides characterized by β-(1,3) glucose bonds or β-(1,6) branched bonds [40]. They are usually derived from macroalgae, especially brown algae, but can also be isolated from microalgae [64]. It has been reported that the average β-glucan content in brown algae ranges from 10 to 30% dry weight. However, the amount of β-glucans derived from algae can be affected by environmental conditions; therefore, they should be kept under control for sustainable productions [65,66]. These complex polysaccharides can maintain blood glucose levels by inhibiting α-amylase, which is involved in carbohydrate metabolism, and prevent the proliferation of tumor cells by inducing cell apoptosis [67]. Some previous studies have indicated that dietary fibers such as β-glucan promote Lactobacillus strain growth by exhibiting prebiotic activity and can maintain intestinal health [68]. In 2020, Gotteland et al. mentioned that the fermentation of β-glucans, especially laminarin, in the large intestine led to an increase in SCFA production and a reduction in the formation of certain harmful metabolites [69].

Laminarin is a storage polysaccharide that is classified as β-glucan and can be naturally derived from both macro- and microalgae, generally brown [65]. It is composed of glucose units and contains both β-(1,3) and β-(1,6) glycosidic bonds [70]. Although the amount of laminarin harvested varies with environmental factors, it can constitute 35% of the dry weight of brown algae [71].

Brown algae species such as *Laminaria* sp., *Saccharina* sp., *Ascophyllum* sp., *Fucus* sp., *Sargassum* sp., and *Undaria* sp. are known for their rich laminarin content and have numerous health benefits: antitumor, anti-inflammatory, antiviral, antioxidant, and anticoagulant activities [72]. In an animal study, laminarin was applied topically to an atopic dermatitis-like lesion [41]. It showed that laminarin can attenuate these lesions by reducing IgE production and mast cell accumulation. This study emphasizes that laminarin may be an effective agent for wound healing. Laminarin, extracted from Laminaria species, modulates gut health and microbiota by exhibiting a prebiotic effect [73]. β-glucans such as laminarin are promising bioactive components for the food and medical industries due to its biodegradable, and biocompatible structure. However, preclinical and clinical studies on nutraceutical and pharmaceutical, and therapeutic effects of these APSs are limited [71]. Therefore, further research is necessary to thoroughly investigate these effects.

#### 2.2.2. Alginate

Alginate is the structural components of the cell walls in brown algae. It exists as divalent salts of alginic acid and forms an intracellular gel-like matrix. Structurally, alginates are linear polysaccharides in which β-D-mannuronic acid (M) and α-L-guluronic acid (G) units are linked by 1→4 glycosidic bonds [45]. *Laminaria hyperborean*, *Macrocystis pyrifera*, *Laminaria digitata*, *Ascophyllum nodosum*, *Sargassum* spp., *Laminaria japonica*, *Ecklonia maxima*, and *Lessonia nigrescens* are the primary algae species utilized for alginate extraction [74]. The % weight of alginate varies from species to species. It is known that it can reach up to 40% of algal dry weight [75].

Alginate is commonly used in the food industry as thickeners and emulsifiers due to their high gelling ability. It is a non-toxic product suitable for innovative applications such as stable emulsions and nanocapsules because it has the ability to cross-link with specific divalent ions [76]. Besides these, as a biodegradable, low-cost biopolymer with low oxygen permeability, it is attracting interest as an eco-friendly and novel alternative for food packaging. Alginate-based nanocomposite films and coatings can increase mechanical strength and extend the shelf life of foods. With these effects, it has great potential for sustainable and resilient packaging solutions [26].

Alginate is an APS that can be used both as a nutraceutical and a pharmaceutical industry. It can reduce inflammation and allergic responses by activating the immune system. Some forms may also have antitumor effects. Additionally, alginate demonstrates positive effects on weight control, glucose regulation, and satiety, making them a potential natural supplement in the fight against obesity and type 2 diabetes [26]. A study analyzed an alginate formulation [77]. It was reported that alginate can increase the feeling of satiety and delay gastric emptying by showing resistance to disintegration in the stomach. Another study comparing the prebiotic effects of sodium alginate with other prebiotics found that sodium alginate significantly increased the number of *Bacteroides* and *Faecalibacterium*. Both of them are beneficial for intestinal health [78].

Despite these beneficial effects to metabolism according to European Food Safety Authority, alginate and its salts (E 400- E 404) are permitted for use in a wide range of foods at the necessary doses. Studies have reported that alginate and derivatives are almost indigestible by humans, are not absorbed in entirety, and are partially fermented by the gut microbiota. Due to the formation of gel-like viscous structure in the stomach contents, intestinal transit may slow down. It has been reported that high doses of alginate (15 g) increase satiety by increasing the viscosity of intestinal contents. Increased viscosity thickens the water layer and slows down the absorption rate of soluble fiber. Which may delay the transit of nutrients in the intestinal lumen. These effects are physiological conditions that may cause sensations such as bloating and constipation. However, the literature does not explicitly classify this as a side effect [79,80].

### 2.3. Extracellular Polysaccharides

Extracellular polysaccharides (EPS) are macromolecules that may be sulfated or not. Degree of sulfation changes depending on environmental conditions. This makes it difficult to classify them directly as sulfated or non-sulfated polysaccharides [81].

EPS are macromolecules that is mainly derived from microalga species [82]. Their major components are mostly polysaccharide’s structure. Various terms are used in the literature for EPS; these include exopolysaccharides, extracellular polysaccharides, and exopolymeric substances. Although these terms are often used interchangeably, they are not always completely equivalent in terms of scope and composition. Some researchers have argued that there is no significant difference between these different terms [83]. The chemical and structural composition of EPSs may vary depending on the algal species and growth conditions. They are secreted outside the cell and play a key role in assisting algae adapt to external stresses including pH, salinity, and toxic substances [83]. A study has indicated that the EPS additive provides protection against environmental stresses like light stress and dryness [84]. With these effects, it is thought that it can be used in food products.

EPSs are also a source of important biological activities that can function as antiviral, anticancer, antioxidant, anticoagulant, and anti-inflammatory agents [85]. They have potential application in the food products. Plus, they can be applied in the animal feed, biomedical, and pharmaceutical industries. Due to their coagulation and heavy metal removal properties, they can also be used in environmental protection areas. According to researchers, they are considered promising biological products in both biomass harvesting and wastewater treatment [83]. Hyrslova et al. (2021) analyzed the health effects of combining Chlorella vulgaris, known for its high EPS content, with *Bifidobacterium animalis* subsp. lactis BB-12 [86]. The study showed a reduction in triglyceride levels in the serum, liver, and hearts of mice. The same study suggested that including *C. vulgaris* in functional food products in combination with bifidobacteria can help preserve the viability of bifidobacteria. Also, non-digestible EPSs exhibit prebiotic activity and modulate gut health and microbiota balance. The long-chain polysaccharides secreted by microalgae during growth resist digestive enzymes. They pass into the colon and act as prebiotics, supporting the gut microbiota [24]. When all of these effects are considered together, they can be a good source for a functional food industry.

## 3. Fermentation by Gut Microbiota

A healthy gut microbiome is made up of a balanced community of various microorganisms that are vital to overall human health. This balance refers to a status where beneficial bacteria are dominant and potentially harmful species are kept under control [87]. In this system, beneficial bacteria such as *Bifidobacterium* and *Lactobacillus* play a key role. These species protect intestinal integrity and regulate the immune response by preventing the proliferation of harmful microorganisms. On the other hand, certain strains of *Escherichia coli* or pathogens such as *Helicobacter pylori* can cause dysbiosis by proliferating excessively and lead to chronic inflammation or gastrointestinal diseases [88].

Prebiotics are indigestible fibers that support the growth and activity of particularly beneficial bacteria that provide health benefits. They show great promise in changing the composition and function of the gut microbiota when consumed in adequate amounts [88]. Prebiotics are fermented by probiotics in the colon, increasing SCFA production and thereby supporting intestinal health. Butyric acid is the main source of energy for colonocytes, with 70–80% of their energy requirement being supplied by butyric acid oxidation [89].

**Figure 2:**
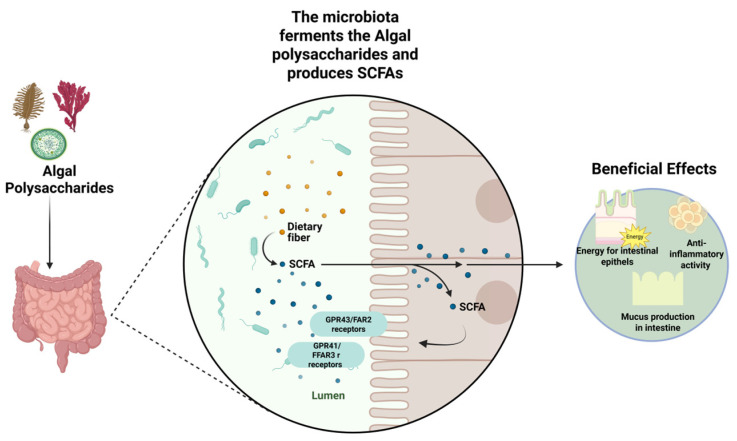
Algal Polysaccharides Fermentation by Gut microbiota. APSs, indigestible by human enzymes, are metabolized by gut microbiota in the colon, leading to the production of SCFAs. These SCFAs contribute to gut health, enhance immune function, and support metabolic balance.

APSs are resistant to digestion in the stomach and small intestine [30]. These components, defined as prebiotics, are indigestible food components that are metabolized by microorganisms in the large intestine, modulating the composition or activity of the intestinal microbiota and providing a beneficial physiological effect on the host. Some water-soluble polysaccharides found in algae are fermented by gut microbiota, leading to an increase in SCFA production (Figure 2) [25]. The gut microbiota has specialized enzymes that can digest these complex carbohydrates. For example, the *Bacteriodes plebeius* bacterium isolated from the intestines of regular Japanese seaweed consumers produce a β-agarase enzyme. The enzyme is specific to the agarose and porphyran components of red algae. This enzyme breaks the β-1,4 bonds that form agarose, creating neoagaro-oligosaccharides [90]. Similarly, laminarin is effectively fermented by laminarinase and β-glucosidase enzymes from certain species such as *Bacteriodes thetaiotaomicron*, *Bacteriodes distasonis*, and *Bacteriodes fragilis*. For the degradation of carrageenan *Bacteriodes xylanisolvens* cleaes carrageenan oligosaccharides with β-carragenase. Moreover, these polysaccharides contain sulfate groups, sulfatase that remove sulfate plays a crucial role. The sulfatese activity constitutes the first step in the hydrolysis of sulfated APSs such as fucoidan and ulvan [91]. Recent studies on ulvan hydrolysis have shown that certain *Bacteriodes* species in the human gut microbiome carry PL24 family lyase homologous capable of cleaving ulvan [92]. This finding suggests that the human microbiome may also possess enzymatic agents that can break down ulvan.

Polysaccharides such as laminarin and alginate are also metabolized by *Bacteroides* species. They can break down and metabolize APSs through specific Polysaccharide Utilization Loci (PUL) clusters [93]. For example, the *Bacteroides ovatus* G19 strain secretes both α-1,4-guluronanlyase and β-1,4-mannuronanlyase [91]. Similarly, a PL17subfamily exomannuronanelyase identified in the genome of *Bacteroides egerthii* is involved in alginate depolymerization [93]. Laminarins is degraded by laminarinase and β-glucosidase activities in certain *Bacteroides* species. In these bacteria, laminarinase activity is induced in the presence of laminarin. They can break down laminarin and oligosaccharides into glucose monomers [94].

SCFAs as a result of fermentation exert their effects at the receptor level. They signal G protein-coupled receptors. For instance, acetate and propionate activate GPR43/FFAR2 and GPR41/FFAR3, while butyrate activates GPR109 suppressing the NF-κB pathway and promoting regulatory T cell differentiations [95]. Thus, SCFAs produced by the microbial fermentation of APS in the intestine contribute to maintaining intestinal health by activating mechanisms that strengthen the epithelial barrier, increase immun tolerance, stimulate IL-10 and sIgA production, and reduce inflammation.

APSs also reduce cholesterol and glucose levels and modulate immune responses. They can assist in weight control by slowing digestion due to their viscosity. Polysaccharides, including exopolysaccharides, alginates, and carrageenans isolated from algae, can be effective prebiotics since they cannot be digested by the digestive system [25]. The effects of Chlorella pyrenoidosa, which contains multiple glycosidic bonds in its structure, on healthy individuals and celiac patients have been investigated [96]. The addition of *C. pyrenoidosa* causes an increase in *Prevotella*, *Ruminococcus*, and *Faecalibacterium* species in healthy individuals, while in celiac individuals, *Faecalibacterium*, *Bifidobacterium*, and *Megasphaera* increase and Enterobacteriaceae decrease. In addition, a significant increase in propionate and butyrate levels was observed.

Polysaccharides derived from edible algae such as *Enteromorpha clathrata* are not easily absorbed in the gastrointestinal tract after oral administration due to their large molecular weights. Therefore, when they reach the cecum and distal colon, they can be fermented by certain microorganisms in the human intestine [97]. A dietary polysaccharide derived from *Enteromorpha clathrata* significantly improved gut dysbiosis in mice fed a high-fat diet, reshaping the structure of the gut microbiota [98]. In particular, it increased the abundance of beneficial bacteria such as *Eubacterium xylanophilum* and *Prevotellaceae*. A decrease in the number of opportunistic pathogens, including *Mucispirillum*, *Desulfobacterota*, and *Alphaproteobacteria*, was also observed.

Laminarin supports the growth of beneficial bacteria, including Bifidobacterium and Lactobacillus. Also, it can inhibit the growth of pathogens [99]. In a study conducted on humans, sodium alginate was observed to significantly increase abundance of Bifidobacteria and decrease number of pathogenic strains such as Enterobacteriaceae by affecting the composition and metabolic activity of the fecal microbiota [100]. It significantly reduced the levels of putrefactive products such as fecal sulfur, phenol, p-cresol, and indole, while significantly increasing the levels of SCFAs. A noticeable reduction in fecal odor was also recorded.

In a comparative in vitro study conducted by Bai et al. (2017), it was found that both *Pseudomonas aeruginosa* and alginate were completely degraded by fecal bacteria isolated from volunteers [101]. *Bacteroides xylanisolvens* strains capable of degrading alginate were identified. Enrichment of the Bacteroides genus was observed in fermentations with both alginate types. In addition, significantly higher production of SCFA was detected in both alginate groups compared to the starch-added medium. According to Ai et al. alginate derived from *Laminaria japonica* reaches the large intestine without being digested by the human upper digestive system and is metabolized by the human intestinal microbiota [102]. The study suggests that alginate increases the relative abundance of beneficial bacteria such as *Bacteroidetes* and *Faecalibacterium* while reducing the levels of *Firmicutes* and *Proteobacteria*. Researchers believe that this modulation may improve obesity-related gut dysbiosis by increasing SCFA production. A recent study mentioned that fucoidan has the ability to modulate the gut microbiota in rotenone-induced Parkinson’s disease mice [22]. It reduced the abundance of *Akkermansia muciniphila* and *Lactobacillus johnsonii* while increasing the number of the probiotic *Lactobacillus murinus*. These changes contributed to the maintenance of the intestinal balance and the reduction in the inflammation. On the other hand, in a study on chicks indicated that nutritional supplementation with laminarin and fucoidan extracts have no effects on *Campylobacter jejuni* or Lactobacillus populations [103].

Fucoidan extracted from *Undaria pinnatifida* exerted a prebiotic effect on *Lactobacillus rhamnosus* under in vitro fermentation conditions. It significantly increased the number and growth of probiotics at appropriate concentrations [40]. In addition, it has been observed that the antibacterial activity of this probiotic against pathogens, including *Staphylococcus aureus*, *Escherichia coli*, and *Enterococcus faecalis*. A study on agar and its derivatives demonstrated that it can be fermented by *Bifidobacterium infantis* and other HMO-using probiotics [104]. Interestingly, the same study also revealed an anticancer activity of agar. It suppressed the growth of HCT-116 colon cancer cells and induced apoptosis. Based on these findings, it can be said that APSs can protect intestinal health while also offering multiple therapeutic activities. Also, APSs can modulate intestinal morphology, increase intestinal secretion, and thicken the physical barrier of the intestine. For example, porphyran modulated gut morphology by exerting prebiotic activity in a study conducted on mice [105]. It increased SCFA products significantly, and the intestinal epithelial cells of mice are arranged in a more regular and compact manner. Also, the mucosal thickness has increased. Studies on APSs have shown that certain polysaccharides exert prebiotic properties and protect intestinal health (Table 1). They can provide protection from certain chronic diseases by regulating gastrointestinal tract and gut microbiome [99].

As shown in the table above (Table 1) APSs increase the number of beneficial bacteria in the gut microbiota while helping reduce the concentration of pathogens. This plays a key role in maintaining gut health by affecting SCFA production.

## 4. Health Benefits

Micro- and macroalgae are attracting considerable interest as sustainable and natural resources in both scientific and industrial fields due to their positive impacts on health [112]. To date, numerous studies have been conducted on the bioactive properties of algae. They provide protection against numerous diseases, including cardiovascular diseases, diabetes, and obesity. They also possess neuroprotective, anti-hypertensive, anticancer, antioxidant, antiviral, anti-inflammatory, and immunomodulatory effects [113].

One of the most important characteristics of algae is their high polysaccharide content. APSs possess many biological activities. Some of these properties include anticancer, antiviral, antioxidant, immunomodulatory, and antidiabetic properties. They are becoming increasingly popular as potential sources of natural pharmaceuticals [63]. Due to their various structural features and potent bioactivities, APSs have garnered significant attention across multiple disciplines. The main fields are food science, human health, biomedicine, biotechnology, cosmetics, animal nutrition, and pharmaceutical development [30]

### 4.1. Anti-Inflammatory and Barrier Function Improvement Properties

Many APSs have the potential to reduce inflammation and protect barrier integrity both in the intestine and at the systemic level. For example, fucoidan has improved intestinal inflammation and mucosal damage in mice with Parkinson’s symptoms [22]. Yang and colleagues reported that it supports the intestinal barrier by increasing the expression of tight junction proteins ZO-1 and occludin. Additionally, it significantly reduced systemic inflammation by lowering blood levels of LPS, TNF-α, and IL-1β and was found to inhibit neuroinflammation through the inhibition of the LPS/TLR4/NF-κB signaling pathway. These effects were highlighted as contributing to reduced dopaminergic neuron damage and improved motor function.

In an adult zebrafish intestinal inflammation model, the anti-inflammatory potential of alginate oligosaccharides and β-glucan derived from algae was investigated [114]. AOS, which has a higher low molecular weight fraction, reduced the expression of inflammatory genes, increased the expression of antioxidant genes, improved intestinal histomorphology by increasing the number of goblet cells and villus length, and increased the level of 2-hydroxybutyric acid, which is SCFA. The β-glucan diet suppressed certain immune genes associated with endopeptidase activity and proteolysis.

In another study conducted on chicks, laminarin supplementation increased small intestine villus size and villus height [103]. It demonstrated positive effects on the intestinal barrier. and immune response. These effects are associated with increased gene expression of the tight junction protein CLDN1 and increased expression of TLR4 and TNF-α. Porphyran, notable for its antibacterial and antiviral properties. On the other hand, it exhibited prebiotic effects in mice. It increased SCFA production and improved intestinal epithelial morphology [105].

### 4.2. Metabolic Management and Effects on Weight

APSs may be effective in weight management and improving metabolic parameters. In vitro and in vivo studies on mice using sodium alginate have found that it forms a gel in the stomach [20]. In this way it reduced the hydrolysis rate of dextrin and whey protein isolate and encapsulated nutrients to slow digestion. In short-term consumption, it prolonged gastric emptying time reduced food intake within 4 h. Additionally, it inhibited carbohydrate digestion and absorption, slowed the rate at which blood sugar enters the bloodstream. In long-term consumption, it reduced food intake and body weight, leading to a significant decrease in fasting blood sugar levels. It was also observed that it increased the length of the small intestine in mice to enhance nutrient absorption. The study suggests that sodium alginate may potentially be effective in the treatment of metabolic syndrome and obesity in humans.

Similarly, oral administration of polysaccharides obtained from *Enteromorpha clathrata* dramatically reduced body weight and lowered serum triglyceride and cholesterol levels in mice fed a high-fat diet [98]. This anti-obesity effect has been reported to be associated with its ability to modulate the gut microbiota, particularly by increasing the abundance of beneficial bacteria such as *Eubacterium xylanophilum*, which produces butyrate. In a study on chicks, feed intake and total weight gain increased in chicks fed diets supplemented with laminarin or laminarin/fucoidan [103]. Laminarin and laminarin/fucoidan diets increased small intestine villus size, while only the laminarin diet increased villus height. These effects promote growth in chicks and are distinct from weight-reducing effects.

Moreover, sulfated EPS from *Chlorella* have demonstrated anti-α-d-glucosidase activity, indicating their antihyperglycemic potential [115]. This enzymatic inhibition further supports the role of APSs in regulating glucose levels and managing metabolic disorders.

### 4.3. Antioxidant Properties

Seaweed compounds have strong antioxidant capacity. Polysaccharides isolated from *Sargassum fulvellum* have strong in vitro and in vivo antioxidant activity [116]. They have been found to effectively scavenge DPPH, hydroxyl, and alkyl radicals, exhibiting the strongest alkyl radical scavenging activity. The polysaccharides significantly reduced intracellular reactive oxygen species (ROS) in Vero cells in a dose-dependent manner and improved cell viability. Additionally, they reduced apoptotic body formation by affecting proteins that regulate apoptosis. In the zebrafish model, it demonstrated a strong protective effect by increasing survival rates against AAPH-induced oxidative stress, reducing heart rate, and decreasing ROS, cell death, and lipid peroxidation levels. The antioxidant effects of EPS, another APS, have been studied, and it has been found to have a significantly high ability to scavenge hydroxyl radicals [117]

### 4.4. Antimicrobial Properties

Carrageenan exerted potent antiviral activity against many enveloped viruses, including influenza A and B viruses, SARS-CoV-2, rhinoviruses, and herpes simplex virus [47,118]. Its subtypes—iota-, kappa-, and lambda-carrageenan—prevent viruses from binding to cell surface receptors and entering cells. It may also affect other steps in viral replication [119]. Lambda-carrageenan reduced body weight loss and increases survival rates in influenza A-infected mice [118]. Nasal sprays containing iota-carrageenan reduced common cold symptoms and combined with oral ivermectin, significantly reduced COVID-19 incidence among healthcare workers [47,118,119].

Studies conducted with fucoidan, which is also sulfated polysaccharide, have emphasized its antimicrobial effects. The antimicrobial and antibiofilm effects of fucoidan isolated from *Fucus vesiculosus* on dental plaque bacteria were investigated, and it was reported that fucoidan showed significant inhibition of growth, including *Streptococcus mutans* [120]. At concentrations above 250 µg mL^−1^, *S. mutans* and *Streptococcus sobrinus* completely inhibited both biofilm formation and planktonic cell growth.

### 4.5. Immunomodulatory and Anticancer Properties

In addition to their other effects, APSs exhibit promising anticancer and immunomodulatory effects. Lambda-carrageenan showed potential as an anti-tumor agent and cancer vaccine adjuvant by enhancing immune cell infiltration and pro-inflammatory cytokine secretion, without causing significant toxicity [23]. EPS, another sulfated polysaccharide obtained from *Coelastrella* sp., has shown anticancer activity by reducing the survival rate in cervical (HeLa) and breast (MCF-7) cancer cell lines [121]. Other evidence has verified the idea that APSs exhibit anticancer and antiproliferative activity by regulating immune responses [122,123]

### 4.6. Anticoagulation—Antithrombotic and Antiplatelet Properties

APSs have also demonstrated promising anticoagulant activities in recent studies. Sulfated APSs in particular, have gained attention as potential lead compounds for the development of novel anticoagulant drugs [124]. Faggio et al. (2016) reported that sulfated polysaccharides extracted from *Ulva fasciata* and *Agardhiella subulata* can act as effective anticoagulants [125]. These extracts significantly prolonged both prothrombin time (PT) and activated partial thromboplastin time (APTT), indicating inhibition of blood clotting. Similar results were confirmed in a more recent study [126].

In another investigation, sulfated polysaccharides isolated from *Chaetomorpha aerea* showed strong anticoagulant effects in both in vitro and in vivo experiments [126]. While they had an effect on APTT and thrombin time (TT), they had very low effect on PT. These observations suggest that the anticoagulant mechanism involves inhibition of intrinsic coagulation factors (XII, XI, IX, VIII) and enhancement of thrombin inhibition through heparin cofactor II (HCII) or antithrombin III (ATIII).

On the other hand, polysaccharides obtained from *Gelidiella acerosa* have strong antiplatelet effects and significant antithrombotic effects in rats. Despite that they have low anticoagulant activity. The most important finding is that, unlike heparin, these effects occur without significantly increasing the risk of bleeding. This result makes these polysaccharides a safe antithrombotic candidate [36]. But these effects need to be confirmed by future studies. Based on these results, it can be said that polysaccharides obtained from algae are a promising source for the development of new antithrombotic drugs.

### 4.7. Neuroprotective Properties

Polysaccharides extracted from algae can prevent neuroinflammation and exhibit neuroprotective properties by maintaining gut microbiota balance (Table 2). Fucoidan derived from *Laminaria japonica* was examined in vivo in mice with a Parkinson’s disease model induced by rotenone (ROT) [22]. It significantly improved ROT-induced parkinsonism by regulating the gut–brain axis. Notably, it reduced neuroinflammation, preventing damage to dopaminergic neurons. In this connection it improved motor function disorder and walking abnormalities observed in mice. Research on this topic is still scarce, so the role of APS in the brain–gut axis remains unclear. Future studies are needed to clarify these effects.

Sulfated polysaccharides obtained from *Botryocladia occidentalis* have shown similar results [127]. They exerted neuroprotective properties against gp120 and Tat in a dose-dependent manner. However, they provided protection through different mechanisms. Surface plasmon resonance analyses have revealed that these polysaccharides bind to these proteins at the nano level. They show a high affinity for the Tat protein. These polysaccharides were effective against both HIV proteins alone and in combination. Additionally, they exhibited anticoagulant activity. The experimental data suggest these polysaccharides may be effective in reducing HIV-1-induced neurotoxicity through direct molecular interactions and represent a promising therapeutic candidate for neuroHIV treatment. When these findings are taken together, APSs may have a direct or indirect neuroprotective effect.

**Table 2:** Bioactivities of polysaccharides obtained from various types of algae.

Polysaccharides	Algae	Method	Activity	References
Fucoidan	*Saccharina japonica*	in vitro (ABTS, FRAP tests) in vivo (H22 tumor mice)	The antioxidant capacity was found to be 1.02 mg TE/g and 5.39 mg TE/g, respectively, based on the results of the ABTS and FRAP tests. A 42.93% reduction in tumor volume was observed in the H22 tumor mouse model.	[122]
Fucoidan	*Laminaria japonica*	in vivo (Rotenone-induced Parkinson’s disease in mice)	It has reduced neuroinflammation and prevented damage to dopaminergic neurons. Parkinson’s disease in mice has significantly improved ROT-induced Parkinsonism by regulating the microbiota-gut–brain axis.	[22]
EPS	*Coelastrella* sp. BGV	in vitro (human cell line)	The MTT test showed that it reduced cell viability in HeLa (cervical cancer) and MCF-7 (breast cancer) cell lines.	[121]
Sulfated EPS	*Chlorella* sp.	in vitro anti-α-d-Glucosidase activity (Human-derived enzyme)	It inhibited the α-d-glucosidase enzyme by 80.94 ± 0.01% and the IC_50_ value was determined to be 4.31 ± 0.20 mg/mL.	[115]
Alginate oligosaccharides (AOS) and β-glucans	*Laminaria* (AOS), *Euglena gracilis* (β-glucans)	in vivo (zebrafish)	AOS suppressed soy-induced intestinal inflammation, increasing goblet cell numbers, extending villus length, and regulating anti-inflammatory gene expression. β-glucan suppressed endopeptidase activity and proteolysis-related immune genes in zebrafish, exhibiting tissue damage-reducing and antioxidant effects.	[114]
Sulfated polysaccharides	*Chaetomorpha aerea*	in vitro (APTT, TT, PT, and fibrinogen level) in vivo (rats)	Inhibits factors XII, XI, IX, and VIII in the intrinsic pathway and stops the coagulation process by suppressing the activity of thrombin and factor Xa via antithrombin III and heparin cofactor II.	[126]
Laminarin	*Laminaria digitata*	in vivo (mice)	In atopic dermatitis-like skin lesions, laminarin alleviates inflammation and modulates immune responses by suppressing IgE hyperproduction, mast cell infiltration, and the release of pro-inflammatory cytokines such as IL-1β, TNF-α, MCP-1, and MIP-1α.	[41]
EPS	*Botryococcus braunii* SCS-1905	in vitro (ABTS, hydroxyl, DPPH, superoxide anion radical scavenging assay)	It suppressed the formation of hydroxyl radicals by chelating Fe^2+^ ions.	[117]
Fucoidan	*Saccharina dentigera*	in vitro (soft agar assay)	It exhibited a pronounced and non-toxic anticancer activity by inhibiting colony formation in human small intestine adenocarcinoma (HuTu 80), malignant melanoma (RPMI-7951), and colorectal adenocarcinoma (HCT-116) cells.	[128]
Sulfated Galactan	*Botryocladia occidentalis*	in vitro (glial cells from mice)	HIV-1 proteins Tat and gp120 have shown neuroprotective activity.	[127]
Iota-Carrageenan	Red algae *	in vitro (human Calu-3 cells)	In Calu-3, it inhibited SARS-CoV-2 replication in a dose-dependent manner; particularly when administered prior to infection, it demonstrated a potent antiviral effect by blocking the viral entry phase.	[119]
Agar and derivatives (3,6-Anhidro-L-galactose)	Red algae *	in vitro (HCT-116 human colon cancer cells and CCD-18Co normal colon fibroblasts)	It significantly inhibited the proliferation of HCT-116 cells and induced apoptosis, but did not show any toxic effects on CCD-18Co cells.	[104]
Lambda-Carrageenan	Red algae *	in vitro (Human Calu-3 cells)	It has been shown to inhibit SARS-CoV-2 replication in a dose-dependent manner, particularly when administered prior to infection, demonstrating a potent antiviral effect by blocking viral entry.	[118]
Fucoidan	*Undaria pinnatifida*, *Fucus vesiculosus*, *Macrocystis pyrifera*, *Ascophyllum nodosum*, *Laminaria japonica*	in vitro (Human peripheral blood mononuclear cells, THP-1)	It has inhibited the production of pro-inflammatory cytokines such as TNF-α, IL-1β, and IL-6.	[67]
β-1,3-glucans	*Euglena gracilis*	in vitro (Portunus trituberculatus hemocytes)	It has been shown to stimulate the immune system by increasing phenol oxidase, lysozyme, acid phosphatase, superoxide anion production, and superoxide dismutase activity.	[129]
Polysaccharides derived from *Enteromorpha clathrata*	*Enteromorpha clathrata*	in vivo (mice fed a high-fat diet)	It has been shown to alleviate obesity by improving intestinal dysbiosis.	[98]
Fucoidan	*Saccharina japonica*	in vitro (RAW 264.7 macrophage cell line) in vivo (zebrafish)	LPS-stimulated RAW 264. 7 macrophage cell line, LPS-induced RAW 264.7 macrophages, thereby inhibiting the iNOS, COX-2, MAPK, and NF-κB signaling pathways. In vivo, it demonstrated a potent anti-inflammatory effect by reducing cell death, ROS, and NO production in zebrafish embryos and improving heart and survival rate.	[21]
Alginate	Brown algae	in vitro digestion in vivo (rats)	It delays food digestion by forming a gel in the stomach, increases the feeling of satiety in the short term, and reduces food intake, body weight, and blood sugar spikes in the long term.	[20]
Fucoidan	*Fucus evanescens*	in vitro (Vero, MT-4 cells) in vivo (mice)	It has shown antiviral, anti-inflammatory and immunomodulatory effects against HSV-1, HSV-2, ECHO-1, and HIV-1.	[130]
Sulfated polysaccharides	*Sargassum fulvellum*	in vitro (Vero cells) in vivo (zebrafish)	These polysaccharides have been shown to exhibit activities including free radical scavenging, ROS suppression, enhancement of cell viability, prevention of apoptosis, regulation of heart rate, reduction in lipid peroxidation, and prevention of cell death.	[116]
Sulfated polysaccharides	*Bangia fusco-purpurea*	in vitro *(Porcine*, *Saccharomyces cerevisiae Enzyme inhibition assay*	They inhibited α-glucosidase in a concentration-dependent manner.	[19]
Fucoidan	*Fucus vesiculosus*	in vitro (bacterial culture)	It has shown significant antimicrobial and antibiofilm activity against dental plaque bacteria; in particular, it completely inhibited biofilm formation and planktonic cell growth of *Streptococcus mutans* and *Streptococcus sobrinus* at concentrations above 250 µg/mL.	[120]
Laminarin, Fucoidan	*Laminaria digitata*	in vivo (broiler chicks)	They have improved growth performance in broiler chicks, improved intestinal villus architecture, and modulated the immune response, but has not affected *Campylobacter jejuni* colonization.	[103]
Fucoidan	*Fucus vesiculosus*	in vitro (Huh-7, SNU-761, SNU-3058 cell line) in vivo (BALB/c nude mice)	It has reduced proliferation in hepatocellular carcinoma cells by suppressing ID-1 and inhibited invasion and metastasis in both in vitro and in vivo models.	[131]
Sulfated Polysaccharides	*Ulva fasciata*, *Agardhiella subulata*	in vitro (human blood samples)	They prevent blood clotting by prolonging prothrombin time (PT) and activated partial thromboplastin time (APTT) durations.	[125]
Lambda-Carrageenan	Red algae *	in vivo (mice B16-F10, 4T1, E.G7-OVA cell lines)	When administered by intratumoral injection in B16-F10 melanoma and 4T1 breast tumor models in mice, it suppressed tumor growth and increased the activation of M1 macrophages, dendritic cells, and CD4^+^/CD8^+^ T cells.	[23]
Fucoidan	*Undaria pinnatifida*	in vitro (tumor cells MCF-7, A-549, WiDr, Malme-3M, LoVo and normal cells HEK-293, HUVEC and HDFb)	It showed anti-proliferative (cytotoxic) effects in cancer cells, while exhibiting lower toxicity in normal cells.	[123]

* The specific type of algae has not been specified.

As seen in the table (Table 2) above, APSs provide multiple benefits for protecting human health. Therefore, as their benefits are discovered, they are expected to become more important for the medical, pharmaceutical, and functional food industries.

## 5. Extraction, Processing and Formulation

**Figure 3:**
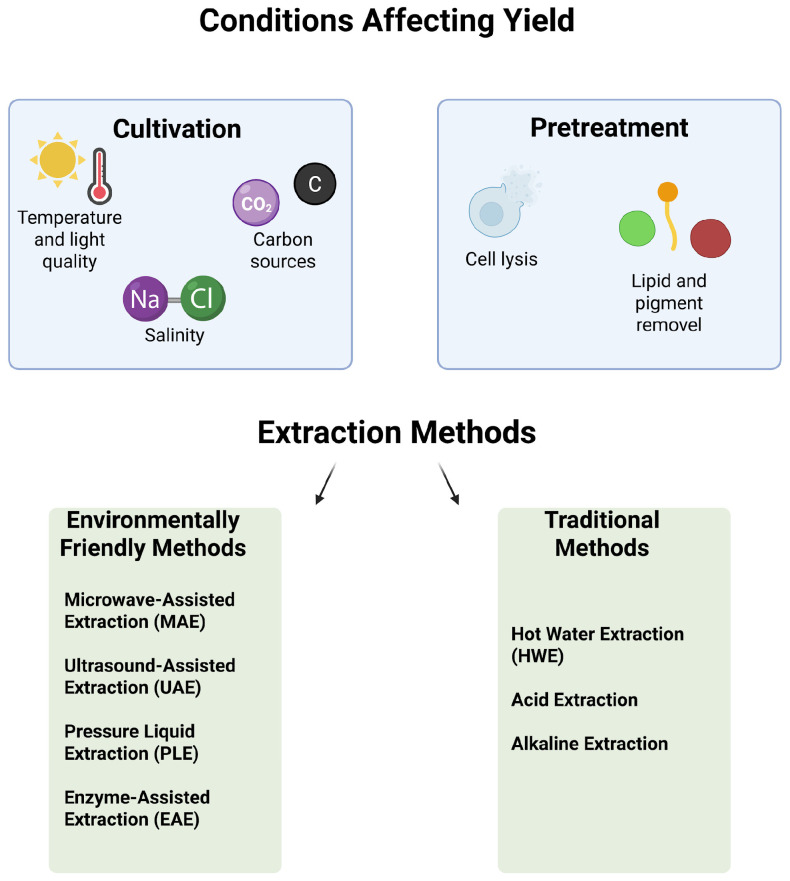
Factors affecting APS yield and extraction methods. The yield and quality of APSs are influenced by environmental conditions such as salinity, temperature and light quality, and carbon sources. Also, APS yield can be affected by pretreatment steps like cell lysis and lipid and pigment removal. Various extraction methods may be used to obtain APS. The method varies depending on the algae used and the polysaccharide to be obtained.

Algae are recognized as a valuable source of bioactive compounds for pharmaceutical and food applications. Although their extraction is often complicated by low polarity and rigid cell walls [132]. Each algal species has a unique polysaccharide profile [133]. In general, brown algae provide compounds such as alginate, fucoidan, and laminarin. Red algae, on the other hand, are primarily a source of carrageenan and agar, while green algae contain ulvan as their main polysaccharide [134]. Both traditional and advanced methods are used for the extraction of APSs. These methods can be chosen depending on the algal species, harvest season, geographical location, and the characteristics of the wanted polysaccharide (Figure 3) [135,136].

### 5.1. Cultivation of Algae for Polysaccharides Yield

Algae cultivation is of significant interest due to the valuable polysaccharides they produce. In marine algae, these polysaccharides can constitute nearly half of the dry biomass, making them a sustainable and industrially important resource [134]. Many species can grow in low-quality water, without consuming freshwater. Moreover, certain microalgae contribute to environmental remediation by absorbing pollutants from wastewater and capturing gas emissions [15,137]. Together, these traits highlight the potential of algae as a sustainable alternative in various bioprocesses. However, the production of algal polysaccharides is not an easy process. Yields are strongly dependent on species and cultivation conditions, which must be carefully optimized [137]. Factors such as water temperature, salinity, currents, and depth can significantly influence production efficiency [135]. Overall, these findings emphasize that controlled growth conditions are critical for achieving high-quality and abundant polysaccharide production.

Environmental factors directly influence growth and photosynthesis rate, cellular metabolism and composition of algae [138]. Limiting nutrients, particularly nitrogen and phosphorus, can enhance polysaccharide accumulation; however, this often comes at the cost of slower algal growth. To address this challenge, a two-stage cultivation strategy has been proposed. In the first stage, biomass is produced under nutrient-rich conditions. The second stage then focuses on increasing polysaccharide content by restricting nutrients. Various nitrogen sources, such as nitrate, urea, and KNO_3_, have been shown to positively influence both EPS synthesis and overall biomass. Additionally, the nitrogen to phosphorus (N/P) ratio plays a critical role and must be carefully optimized to achieve high polysaccharide yields [84,137].

Salt stress can promote polysaccharide production in microalgae. For instance, NaCl-induced stress increased EPS production by 63% in *Microcoleus vaginatus*. Furthermore, increasing freshwater salinity relative to seawater resulted in 3–30 times higher EPS production in *Chlorella* sp. [139]. Salinity significantly changes the biochemical composition of algal cells. Low salinity is beneficial for the accumulation of soluble polysaccharides in macroalgae [140].

Light quality and intensity are important for EPS synthesis. Red light and optimum intensities can increase EPS production in microalgae [139]. Blue and red light can increase algal growth and polysaccharide production. Red light can incorporate carbon into sucrose and starch synthesis pathways, while blue light can significantly reduce sucrose and starch formation in macroalgae [138,141]

Temperature alters the degree of unsaturation of fatty acids in algal cells. Temperatures above optimum can inhibit intracellular lipid synthesis, leading to polysaccharide accumulation. In *Botryococcus braunii*, polysaccharides accumulated while lipid content decreased at 32 °C. However, increased temperatures can also lead to degradation of the starch produced [142,143]. The appropriate temperature range is species-specific. For example, in Anabaena sp., EPS production rises approximately 4–5 times when the temperature is raised to 40–45 °C [83].

### 5.2. Pretreatment Steps and Extraction Methods

The extraction method selected is a key factor in defining the quality and yield of algal polysaccharides [133]. Different algae species and the type of polysaccharide targeted strongly influence this process. External stresses, including growth conditions, harvesting time, and environmental factors, also play an important role [135]. Extraction usually follows a series of essential steps. However, the exact procedure may be different depending on the species and the structural features of the polysaccharide. The chosen method directly shapes yield, purity, molecular weight, and chemical composition, making the decision process highly critical [136]. In recent years, several eco-friendly techniques have gained attention. These include hot water extraction (HWE), ultrasound-assisted extraction (UAE), enzyme-assisted extraction (EAE), and microwave-assisted extraction (MAE). Such methods are described as “green extraction” due to their efficiency, low energy use, and absence of harmful solvents [144]. For sustainable applications, low-cost and environmentally safe strategies remain the preferred choice.

For the efficient extraction of APS, it is crucial that the algae are properly prepared before extraction. The pretreatment stage involves the removal of unwanted substances and the lysis of cells [135].

Cell lysis is an essential stage in APS extraction, as it disrupts or weakens the rigid algal cell wall. This process can be carried out using conventional techniques, but more advanced approaches, such as microwave-assisted methods, are also applied. The main goal is to release intracellular compounds more efficiently. By facilitating this breakdown, cell lysis significantly improves the overall yield of extracted APS [145,146]. In practice, researchers often rely on solvents with varying polarities to extract APSs while preserving their structural integrity. Low-polarity solvents such as chloroform, petroleum ether, and dichloromethane are commonly employed to remove lipids [147]. Yet, not all solvents are considered suitable, since some raise environmental concerns. For instance, Dobrinčić et al. (2020) point out that petroleum ether can be used as a safer option compared to chloroform [135].

At this stage, semi-polar solvents—including ethanol, methanol, and acetone—are typically applied to eliminate pigments. Water is then used to wash away other undesirable algal residues [147]. This step is crucial, as it contributes directly to both extraction yield and the overall quality of the final APS product.

Traditional extraction techniques are typically low-efficiency, time-consuming, and require high energy consumption [133]. HWE is one of the most widely applied methods for obtaining polysaccharides from algae. In this approach, dried algal material is first ground and then extracted with distilled water at high temperatures, usually between 80 and 100 °C, for about three hours [133]. To protect polysaccharide integrity, the pH is maintained close to neutral, around 7. This technique is versatile, as it allows the recovery of different types of polysaccharides. However, a drawback is the presence of residual proteins and pigments, which may reduce purity [135]. Acid-based extraction is often selected for polysaccharide fractions that are soluble under acidic conditions [134]. Dried algae are treated with an acid solution and heated in a water bath at approximately 70 °C for 1–4 h [126]. The acid treatment hydrolyzes algal cell walls and promotes the release of target polysaccharides [135]. Stronger acid concentrations and higher temperatures can improve release but also risk degradation. In some cases, this leads to reduced viscosity, reflecting partial breakdown of the extracted polymers [136]. Alkaline extraction is generally preferred for sulfated polysaccharides such as fucoidan [134]. In this method, the algal cell wall is disrupted and tightly bound sulfated fractions are released. Firstly, the algal powder is dissolved in NaOH for 2–4 h and then neutralized with diluted HCl. However, excessively high pH can cause degradation of the polysaccharides. On the other hand, it can increase gel strength in the production of carrageenan and alginate [136]. Traditional methods for the extraction of polysaccharides from seaweed can be time-consuming, energy-intensive, and inefficient. Also, they carry the risk of degrading the polysaccharides [148]. Recently, to overcome these limitations, many advanced extraction techniques have been developed and applied [135,149]. These methods offer numerous advantages. These methods can increase yield, reduce processing time and cost, reduce solvent use, and preserve the structural integrity of bioactive molecules [148]. For these reasons, it would be more appropriate to choose advanced methods to save time and increase the efficiency of the isolated product. MAE uses electromagnetic radiation on the sample matrix to generate heat directly within the material. This rapid internal heating causes the cell walls to break down effectively. The intracellular compounds to be released into the extraction solvent [135]. When applied to microwave radiation under high pressure, it can also induce the destruction of the cuticular layer [148]. It is considered more efficient than traditional methods and decreases processing time. MAE reduces energy consumption and uses less solvent. These reasons make it an eco-friendly technique [148]. However, some components can be difficult to extract. High temperatures and long extraction times may cause degradation of polysaccharides [133]

Polysaccharides extracted by MAE generally have higher sulfate group concentrations and lower molecular weights. In some cases, higher fucose and lower uronic acid content have been reported [133,150]. Polysaccharides extracted by MAE may exhibit antioxidant and hydroxyl radical scavenging activity, but these activities may decrease with increasing temperature and time. They may also exhibit potential hypoglycemic activity [135,150].

The UAE relies on the propagation of sound frequencies as compression and rarefaction waves on samples. This increases the contact surface between liquid and solid phases and breaks cell walls [148]. It shortens the extraction time and increases yield. Compared to traditional methods, it is effective, environmentally friendly, and economical. Due to these reasons, it has been adopted in the food industry [133]. Compared to traditional maceration methods, it has lower energy consumption and carbon footprint [148]. However, high ultrasonic power can produce hydroxyl radicals that can cause chemical decomposition [135]. The ultrasonic process can cause structural modifications in sulfated polysaccharides. While some studies suggest that higher molecular weight fucoidan is produced, others indicate a decrease in molecular weight [133].

PLE uses high temperatures and pressures to extract compounds in a short time and with less solvent in an oxygen- and light-free environment [135]. High pressure provides the solvent in a liquid state above its normal boiling point. Also, it reduces its viscosity and surface tension [138]. It offers many advantages including high extraction efficiency, reduced solvent consumption, fast and safe extraction [135]. Since water is typically used as the solvent, no toxic byproducts are produced [151]. However, increased pressure and temperature can cause unstable compounds and polysaccharides may degrade. There are few published studies on the use of PLE for algae [148]. On the other hand, PLE can cause an increase in polysaccharide yield. Polysaccharides with lower molecular weight are generally obtained. Also, temperature increase can positively affect sulfate content [135,150].

EAE is considered a cost-effective method since it eliminates the use of hazardous chemicals and organic solvents [125]. The technique can increase both yield and selectivity of specific polysaccharides. However, its wider application is restricted. One key limitation is the narrow substrate specificity of hydrolytic enzymes, which explains why studies on APSs remain relatively few [148]. Nonetheless, EAE has the advantage of reducing polysaccharide molecular weight while maintaining biological activity, and it may also enhance sulfate content and overall molecular characteristics [133].

There are multiple methods that can be used to obtain APS. However, as can be seen from the table below (Table 3), whether the preferred methods are eco-friendly and how they affect yield varies depending on the method used.

## 6. Application in Functional Foods and Nutraceuticals

Marine environments harbor immense biological diversity, encompassing well over half of all known species. Most of these organisms are important producers of complex bioactive compounds, including proteins, lipids, polysaccharides, phenolic molecules, and peptides. These marine-derived biomolecules not only carry out essential biological roles but also exhibit a wide spectrum of functional activities that make them attractive for food and pharmaceutical applications [148]. In response to growing consumer preference for natural, health-oriented products, researchers are now exploring a range of novel functional foods sourced from nature. In this context, algae have emerged as highly promising candidates: they are globally abundant and sustainable and provide rich profiles of nutrients and bioactives. In fact, edible algae have been identified as key sources of functional food ingredients, offering nutritional enrichment and health benefits in food and nutraceutical products [149].

By definition, functional foods are those that confer health advantages beyond traditional nutrition [148]. As discussed in previous sections algae fit this definition well due to their “nutraceutical” qualities. Many algal species accumulate antioxidants, vitamins, minerals and fibers that can positively influence human physiology. Importantly, their beneficial effects are often linked to the polysaccharide components of algae. In support of human health, studies reported that APSs can help alleviate aspects of chronic metabolic disorders, such as improving insulin sensitivity or reducing blood lipids. This can also mitigate conditions including diabetes, cardiovascular diseases, and obesity [150]. These natural marine compounds are attracting great attention for their ability to fortify foods with nutrients and bioactivities [23].

**Figure 4:**
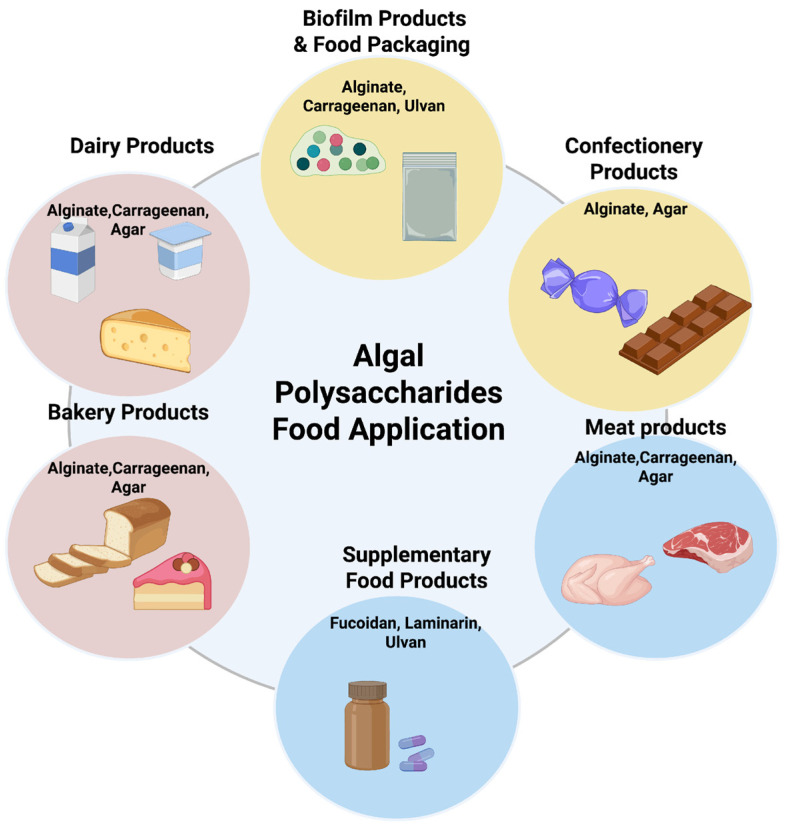
Applications of alga-derived polysaccharides in the food industry. APSs are widely used in food products as thickeners, gelling agents, stabilizers, and emulsifiers.

APSs exhibit important functional properties that are highly valuable in food product development, including gel formation, viscosity enhancement, and stabilization (Figure 4) [30]. Beyond their technological roles, regular intake of APS-based nutraceuticals has been suggested to confer long-term physiological benefits, without detectable adverse effects on human health [151]. Notably, microalgal polysaccharides are currently being explored as promising prebiotic ingredients, offering potential to support gut health when incorporated into functional foods [23].

### 6.1. Applications in Food Products

APSs have been utilized in the food industry for many years, serving not only as bioactive compounds but also as multifunctional ingredients that enhance structure, texture, and sensory qualities. Certain APSs are widely used in the food industry. For instance, carrageenans are frequently incorporated into dairy products, jellies, desserts, and processed meats. They act as reliable thickening and gelling agents, consistently improving texture and appearance, which makes them essential in numerous industrial applications [158]. Alginate is another commonly employed APS, prized for its ability to encapsulate bioactive molecules such as polyphenols and probiotics. It forms stable ionic gels, particularly in the presence of specific cations. Beyond encapsulation, alginate also supports thickening, stabilization, suspension, film formation, and emulsion stabilization. These properties expand its utility across a wide variety of food systems [74].

Agar is also a polysaccharide that has been recognized by the food industry for many years. Moreover, agar and its derivatives extend the role of APSs beyond food formulation. Recently, they have been explored as biodegradable and sustainable packaging alternatives to synthetic plastics, offering both functional and ecological benefits [159]. Additionally, agar-based materials have been studied for their potential anticancer and prebiotic effects, and their importance in the design of functional foods that support human health has been emphasized [104]. Nevertheless, since research on the topics discussed here is quite limited, it is important that future studies confirm these findings. Within functional food research, APSs are increasingly valued for their nutritional and health-promoting properties. For instance, the inclusion of fucoidan at levels of 0.40% and 0.80% in bread formulations has been reported to improve product quality by boosting CO_2_ release during fermentation, enhancing loaf volume, softening crumb texture, increasing pore size, and altering gluten structure. Remarkably, fucoidan retains, and in some cases even strengthens, its antioxidant and anticancer activity after baking, making fucoidan-enriched bread a strong candidate as a functional food [160].

APSs are known to be used to improve the physicochemical properties of strach- based products and expand their potential applications as functional food ingredients. Among these APS laminarin and alginate are prominent. In a study, laminarin was added to starch at ratios of 0–2–4–6–8–10%, and its physicochemical properties were examined [161]. According to this study, 2% laminarin reduced crystallinity and short-range order, while 6% laminarin provided the highest crystallinity and short-range order. Also, the concentration that reduced digestibility the most was 6%. It is thought that the reason for the reduced digestibility is that laminarin forms a barrier on the surface of starch granules, making it difficult for amylases to access the starch chains. Based on these data, it is possible to say that the effect of laminarin on starch is dose-dependent.

On the other hand. The interpenetrating polymer network created using sodium alginate has shown great potential in slowing down starch digestion. Structures with 0.5–1.0–2.0% sodium alginate added were examined, and slight improvements were observed at 0.5% concentration, whereas structural deterioration occurred at 2% because of gelation [162]. The optimum ratio was determined to be 1% sodium alginate addition. Cooking loss predicted Glycemic Index (pGI), and surface stickiness decreased. The product’s (noodle) structural firmness and resistant starch (RS) content increased.

Additionally, in other study, alginate was added to chicken sausage and showed significant antioxidant properties [163]. The addition of 4% sodium alginate notable reduced the values indicative of lipid oxidation, such as free fatty acids (FFA), peroxide value (POV), and 2-thiobarbituric acid (TBA). It improved the microbial quality of chicken sausage. Specifically, it was found that the product could be stored stably for 30 days with 4% of sodium alginate addition. At the same time, it improved the sensory properties of products including color, flavor, tenderness, juiciness and reduced cooking loss. For these reasons, alginate can be a useful additive in meat products such as sausage. Ulvan has shown similar activities in beef sausages.

Applications of APSs also extend to animal nutrition. Fucoidan supplementation in lamb feed has been associated with reduced diarrhea, likely through modulation of gut microbiota, reinforcement of intestinal barrier function, and improvements in antioxidant and immune responses in the colon [164]. In the dairy industry, fucoidan has been successfully incorporated into yogurt, where it improves rheological and textural properties while also supporting probiotic growth and survival, including strains such as *Lactobacillus acidophilus* and *Bifidobacterium lactis* [165]. Microencapsulation approaches employing APSs further enhance the stability and viability of probiotics in dairy products. Saeed et al. (2024) showed that the combination of alginate and carrageenan coatings in cottage cheese improved probiotic survival during storage [166]. The combination also enriched the sensory quality of cheese. Alginate-based hydrogels are also used for encapsulating baker’s yeast. This structure enhances the yeast’s cryotolerance and improves sensory properties of bakery products [167].

Beyond bioactive delivery and microbial protection, APSs also play an important role in preservation and quality maintenance. Alboofetileh et al. (2024) shows that ulvan significantly reduced cooking loss and lipid oxidation in beef sausage formulations [168]. This, it can be inferred that certain APS can be used in foods to preserve product quality. Also, it has inhibited microbial growth and increased the moisture content of the product. Sensory evaluations have shown that ulvan at low concentrations (up to 0.5% *w*/*w*) is sensory acceptable in sausage.

The beneficial effects of algae and their polysaccharides on health, when included in the diet, have been clearly demonstrated in both in vivo and in vitro models [22,103,122]. Consequently, APSs could serve as a promising nutraceutical and functional food ingredient. Nevertheless, continued research in this area will make them more recognized and more acceptable by both the food industry and consumers.

As we can see from the table (Table 4), APS have been incorporated into numerous food products and have shown positive effects on products’ quality. Beyond their conventional roles as thickening or emulsifying agents, their potential use as functional food additives remains an active area of research, with the prospect of achieving enhanced benefits through such applications.

The use of algae and APSs in food products face various limitations. Primarily concerning sensory properties, texture and bioavailability. Changes in texture and consistency, taste, smell, and color significantly reduce the use of algae in food and their overall acceptance rate. For example, in beef sausage containing 1% of ulvan extracted with high-concentration enzymes, a significant decrease in sensory scores was observed, in terms of flavor, texture, and overall acceptability compared to the control group [168]. This shows that APSs can alter the original taste of foods, leading to consumer rejection. Similarly, *Laminaria digitata* added to the broiler diet reduced flavor intensity and juiciness in chicken meat [173]. In the context of texture and physical acceptance, APSs addition also affect the products color. For instance, *Laminaria digiata* reduce the redness of chicken breast meat [173], while ulvan increased the lightness and reduces the redness of sausages [168]. On the other hand, fucoidan showed texture improving effect by reducing the hardness and chewiness of bread [160].

The potential bioavailability limitations of algae due to the complex, indigestible cell walls found in the food matrix. Adding 15% *Laminaria digitata* to the broiler diet reduced the animal’s growth performance and notably increased the viscosity of the intestinal content [173]. Thereby hindering the digestion and absorption of nutrients. Additionally, when laminarin is added to wheat starch, it increases the resistant starch (RS) ratio. Thus, reducing the digestibility of starch [161]. This highlights the potential of APSs to slow down the digestion rate of foods.

In brief, algae and APSs could negatively affect the texture, color, and flavor of food matrices. These effects are more common when used at high concentration [49,50]. Furthermore, in their natural state they contain indigestible components that have the potential to reduce the bioavailability of nutrients in animals and possibly in humans.

Based on these studies mentioned above, it is evident that APSs hold significant importance, particularly in the context of functional food products. However, while the application of APSs in foods offers numerous benefits, it may also present certain limitations [163,168]. To overcome these limitations and preserve their advantages, dosage studies should be conducted and optimized. Additionally, the toxicity assessments, along with evaluations of food quality and applicability, should be carried out to develop appropriate applications.

### 6.2. Regulation and Safety Consideration

Microalgae and macroalgae are gaining increasing attention in functional foods due to the bioactive compounds they contain [64]. The US Food and Drug Administration (FDA) has evaluated various species such as *Arthrospira platensis*, *Chlorella protothecoides*, and *Dunaliella bardawil* under the GRAS (Generally Recognized as Safe) category and has stated that they are suitable for human consumption [181,182,183]. The European Food Safety Authority (EFSA) has similarly classified species such as *Chlorella vulgaris*, *Ulva lactuca*, and *Porphyra tenera* as “traditional foods” and excluded them from the scope of Novel Foods [184,185,186]. Also, certain species components such as *Schizochytrium* sp. oil and *Chylamydomonas reinhardtii* powder considered as novel food according to EFSA [187,188].

While algae are recognized as a new potential food source or ingredient in the European Union (EU), the number of macroalgae species approved for marketing under EU food laws remains limited [185]. Currently, only a small number of algae species have GRAS status and can be sold for direct human consumption [189]. All other algae and their extracts are subject to the Novel Food Regulation [190].

When it comes to microalgae, the picture is more complex. *Tetraselmis chuii* and *Chlamydomonas reinhardtii* (dry biomass form) have been defined as “novel foods” by EFSA and are legally permitted for use in the EU market in accordance with scientific opinions, but neither has received GRAS approval from the FDA [191]. Despite that, DHA oil obtained from *Schizochytrium* sp. has been approved by both EFSA and the FDA [187,192]. Species that have achieved a major level of acceptance, such as *A. platensis* (Spirulina) and *Chlorella vulgaris* [181,183]. They are also considered to be traditional foods by EFSA [181]. *Euglena gracilis*, although less well-known, has been classified as a ‘Novel Food’ in the EU but has been recognized as GRAS by the FDA [190].

In Japan, seaweed flour, oil, phytopigments, and extracts are classified as natural foods. For example, Chlorella extract is used as natural flavorings and colorings. Do not require special permits or limits [193]. China’s used, and consumption of seaweeds is similar to Japan. Seaweed and seaweed extracts are widely accepted as edible natural food [194].

These assessments show that although some algae species are approved and considered safe by EFSA and FDA, others remain limited to region-specific use. Nevertheless, algae with rich bioactive profiles continue to gain importance as promising components in functional foods and dietary supplements [195,196]

While this is the case for algae, APSs such as agar (E406), carrageenans (E407/407a), and alginate (E401) are approval as stabilizers and emulsifiers in food. They are generally considered safe. However, since these long-chain polysaccharides cannot be digested by humans. They behave like dietary fiber. This may limit their systematic bioavailability and require optimization for nutritional applications [197]. As discussed in previous sections, high concentration of certain APSs may cause bloating, constipation, or have an inflammatory effect. Additionally, some types of algae have the potential to accumulate contaminants such as heavy metals. Regulatory authorities have set consumption limits for certain algal products [197]. In this context, the molecular weight, type, and concentration of APSs are important. For instance, high molecular weight sodium alginate has been shown to limit lipid absorption by reducing intestinal mucosal permeability [198]. In a similar way, both high and low molecular weight alginate forms have been reported to prevent nonsteroidal anti-inflammatory drug induced small intestine damage [199]. However, it should be noted that high doses alginate may adversely affect health by significantly increasing intestinal viscosity, which can cause problems with bowel movements [79,80]. These data demonstrate that the APS structure shapes its biological effects and safety. Therefore, a balanced assessment of bioavailability, molecular properties, and toxicity is required.

While EFSA and FDA permit the use of defined additives such as agar, alginate and carrageenans, they stipulate conditions for their use. In Europe, APSs such as alginate, carrageenan and agar have long been used as food additives [197]. According to EFSA’s re-evaluations, it has been concluded that a numerical Additional Daily Intake (ADI) is not required for alginate and its salts. There is no safety concern at current exposure levels [200]. No toxicological concern has been identified for the agar, and the re-evaluation result indicates that there is no safety issue at the general level of use by the populations. For carrageenan, the EFSA ADI of 75 mg/kg has been provisionally retained. However, EFSA has stated that carrageenan is not recommended for infant formula, But may be acceptable as a complementary food for infants over 6 months of age [201]. In the FDA statements, carrageenan is an additive permitted for use in ’quantum satis’ amounts necessary to impart texture and stability to products [202]. Alginate is defined as a GRAS substance and can be used in certain foods within limits ranging from 0.3 to 10%. Agar is also GRAS and can be used in foods as an antimicrobial and stabilizer in good manufacturing practices up to 0.1% [203].

In China, national food safety standards coded ’GB’ regulate the food applications of these polysaccharides. For example, the GB 1886, 169-2016 standard specifies the properties and areas of use of carrageenan additives [204]. In Japan, the Organic Agriculture Standards (JAS) lists permit the addition of sodium alginate and carrageenan (JAS 1606) [205]. The different regional regulatory framework defines in which food categories and in what quantities APSs can be used. Consequently, before the health effects of APSs can be fully understood, issues such as bioavailability, differences related to molecular structure, and safety, as well as international regulations, must be carefully addressed.

However, according to current regulations, specific APSs such as ulvan, fucoidan, prophyran, and laminarin have not yet been approved as food additives or novel foods by the EFSA, FDA, JAS, or GB. Only a few types of algae containing these polysacchardies have been approved by EFSA and FDA. In China and Japan, the use of algae and its derivatives is already widespread.

Food safety is critical for the use of algae as a new food source in industry. In addition to essential nutrients, algae may contain other substances such as phenolic compounds, pigments, fatty acids, minerals and potentially heavy metals [30]. Therefore, additional clinical and preclinical studies are needed to fully confirm algae as a safe food source. Factors such as bioavailability and bioaccessibility are crucial for the efficacy of food ingredients. In vitro digestion models are required for this assessment. Future studies could expand the range of algae in the food industry by focusing on these factors in addition to the safety of algae in food products.

## 7. Conclusions

Algal polysaccharides are promising compounds in terms of both their health impacts and potent functional food applications. Because of their benefits on gut microbiota, they provide important contributions to supporting digestive health, regulating immune response, and preventing certain metabolic diseases. SCFAs formed as a result of fermentation of APS have a broad spectrum of effects on host’s overall health. Yet, the structure of APS can differ depending on its source. Since the molecular structures, solubility, fermentability, and biological effects of polysaccharides can vary, the intended effects of each compound must be evaluated separately. It will be very important to examine these concerns in future research in order to develop a rational approach to algae consumption.

With people becoming more aware of the nutritional value of the food they consume. Due to this condition, the functional food sector is gaining significant growth. In this field, APSs added to food products are attracting interest as a sustainable and natural alternative. Depending on the dose, they can support health and even be used for therapeutic purposes. However, due to insufficient preclinical and clinical studies, there are limitations such as dosage and side effects. Production standardization, extraction efficiency, consumer acceptance, sensory effects such as taste, and smell must be provided by the necessary institutions and organizations. Most importantly, security assessments and investigations must be provided. In particular, the risk of certain types of algae accumulating heavy metals or toxic compounds depending on environmental conditions. These factors are being closely monitored by the relevant authorities. Low bioavailability can limit potential benefits, the toxicity potential of some species. Especially in cases of prolonged exposure and high doses, a cautious approach is required.

Recent studies have highlighted multiple beneficial effects of algal polysaccharides. They have been shown to modulate gut microbiota composition, enhance SCFA production, and improve digestive health. In addition, evidence points to their antioxidant, anti-inflammatory, and immunomodulatory activities, as well as potential roles in regulating lipid and glucose metabolism. These findings from current research collectively support the use of algal polysaccharides in functional foods, nutraceuticals, and emerging therapeutic applications. Furthermore, the mechanism by which these compounds are utilized in the human body are still not fully understood, posing a major challenge for their safe and effective application. To address this issue, comprehensive in vitro and in vivo studies on toxicity, bioavailability, and metabolic pathways are essential. Once these gaps are bridged, algae- derived polysaccharides are expected to gain a stronger position in both the food and pharmaceutical markets.

In the future, combining algae with fermented food products could offer new opportunities in functional food development. Algae polysaccharides may also be used in foods aimed at supporting specific gut microbiota, in line with personalized nutrition plans. Due to their potential health benefits, they can be utilized as nutraceuticals, or dietary supplements. In the near future, it is considered that they could be used as drugs in the pharmaceutical industry. Researchers can alter the polysaccharide structure of algae in many ways for more convenient applications. The molecular structure of polysaccharides can be modified, and solubility and fermentation yield can be increased. Eco-friendly, high-yield extraction techniques, along with product formulations that have better taste and texture, are likely to encourage consumer acceptance. As more clinical studies are conducted, it is expected that regulatory agencies may approve these polysaccharides, leading to wider use in the global functional food market.

Furthermore, it is not known by which mechanisms these compounds are used in the human body. This poses a major problem. In vitro and in vivo toxicology, bioavailability and bioavailability studies are necessary to solve this problem. When these conditions are overcome algae can find more space in the market.

## Figures and Tables

**Table 1:** The effect of APSs on gut microbiota and SCFA production.

Polysaccharides	Method	Microbiota Changes	SCFA Production Changes	References
Fucoidan	in vitro fermentation	*Bifidobacterium*, *Lactobacillus*, *Faecalibacterium*, *Prevotella*, *Megamonas* increase *Escherichia-Shigella*, *Klebsiella*, *Bilophila* decrease	Acetate, propionate increase Butyrate, isobutyrate, valerate, isovalerate decrease ***	[18]
Ulvan	in vitro fermentation	*Bacteroides*, *Parabacteroides*, *Faecalibacterium* increase *Prevotella*, *Blautia*, *Ruminococcus* decrease	SCFA levels increase especially acetate, propionate, butyrate ***	[106]
Agar and derivatives	in vitro fermentation	*Lactobacillus plantarum*, *L. acidophilus*, *L. casei increase* *Escherichia coli*, *Bacillus cereus decrease*	An indirect increase in acetate, propionate, and butyrate levels is expected ***	[62]
Polysaccharides from *Enteromorpha clathrate* *	in vitro fermetation (ulcerative colitis fecal inocula).	*Bacteroides thetaiotaomicron*, *B. ovatus*, *B. uniformis*, *Blautia* spp., *Parabacteroides* spp. (anti-colitic) increase *Escherichia-Shigella*, *Enterococcus* decrease	Acetate levels increase Lactate levels decrease ***	[97]
Laminarin	in vivo (Juvenile spotted seabass)	*Lactobacillus*, *Klebsiella*, *Proteobacteria* increase *Bacillus* decreases	SCFA levels increase **	[73]
Porphyran	in vivo (healthy mice)	*Akkermansia*, *Rikenella*, *Coprococcus*, *Lachnospira*, *Roseburia* increase *Proteus*, *Shigella*, *Anaerofustis* decrease	SCFA levels increase especially acetate and butyrate ***	[105]
Fucoidan and Kappa-Carraheenan	in vivo (rats showing Alzheimer symptoms)	*Akkermansia muciniphila* increase *Peptostreptococcaceae* decrease	Acetate 0.74\pm 0.02 mM Butyrate 0.17\pm 0.003 mM Total SCFA 1.22\pm 0.02 mM	[107]
Lambda Carrageenan	In vivo (rats showing Alzheimer symptoms)	*Akkermensia muciniphila* increse	Total SCFA 1.20\pm 0.02 mM Butyrate 0.17\pm 0.002 mM	[107]
Alginate	in vitro fermentation	*Bacteroides xylanisolvens*, *Faecalibacterium*, *Prevotella copri* increase (potentiallly)	Acetate levels increase ***	[108]
Laminarin	In vitro fermentation	*Erysipelatoclostridium ramosum Bacteriodes uniformis*, *Roseburia faecis*, *Roseburia inulinivorans* increase (potentially)	SCFA levels increase **	[108]
*Chlorella pyrenoidosa* polysaccharides	in vitro digestion and fermentation	*Parabacteroides distasonis* (become dominant), *Phascolarctobacterium faecium*, *Faecalibacterium prausnitzii* increase *Escherichia-Shigella*, *Fusobacterium*, *Klebsiella* decrease	Total SCFA 36.076\pm 0.272 mM Acetate 18.968\pm 0.30 mM Propionate 9.617\pm 0.30 mM	[109]
Agar and derivatives	in vitro fermentation	*Bifidobacterium longum* ssp. *infantis* ATCC 15697, ATCC 17930, ATCC 15702, *Bifidobacterium kashiwanohense* DSM 21854 increase	SCFA levels increase especially acetate and lactate	[104]
Laminarin	in vitro fermentation	*Bifidobacteria*, *Bacteriodes* increase	Acetate 85.7 mM Propionate 28.7 mM	[110]
Ulvan	in vitro fermentation	*Bifidobacteria*, *L. Actobacillus* increase	Acetate 59.9 mM Lactate concentration dropped to 11.5 mM.	[110]
Alginate and derivatives	in vitro fermentation	*Bacteroides ovatus*, *Bacteroides xylanisolvens*, *Bacteroides thetaiotaomicron* increase	Total SCFA 78.6\pm 5.9 mM Acetate 41.3\pm 5.8 mM. Also propionate and butyrate levels increase ***	[111]

* Specific polysaccharide not mentioned. ** Specific SCFA product not mentioned. *** SCFA levels are not mentioned quantitatively.

**Table 3:** Method, yield, and ecological impact in the production of alga-derived polysaccharides.

Method	Eco-Friendly	Algae	Polysaccharides	Yield	References
Ultrasound-Assisted Extraction (UAE) (1300 W 60 min)	Yes	*Ulva* spp.	Ulvan	9.29 ± 0.47%	[152]
Ultrasound-Assisted Extraction (UAE) (176 W 15 min)	Yes	*Sargassum* spp.	Alginate	54.20%	[153]
Enzyme-Assisted Extraction (EAE) (35.3% selulase + 34.5% pectinase + 30.2% alkaline protease)	Yes	*Ulva lactuca*	All polysaccharide content	30.14% (optimum enzyme mixture)	[149]
Hot Water Extraction (HWE) (90 °C 4 h)	Yes	*Ulva lactuca*	All polysaccharide content	6.43%	[149]
Enzyme-Assisted Extraction (EAE) (35.3% selulase + 34.5% pectinase + 30.2% alkaline protease) + Ultrasound-Assisted Extraction (UAE) (90 min)	Yes	*Ulva lactuca*	All polysaccharide content	30.14%	[149]
Alkaline Extraction (2.5% Na_2_CO_3_)	No	*P. pavonica*, *S. cinereum*, *D. dichotoma*	Alginate	21.13–24.08%	[154]
Ultrasound-Assisted Extraction (UAE) (150 W approximetely 30 min)	Yes	*Laminaria digitata*	Alginate	~20–25%	[155]
Enzyme-Assisted Extraction (EAE) (Cellulysin 300 U/g) + Ultrasound-Assisted Extraction (UAE) (70 W 40 min9	Yes	*Ulva fenestrata*	Ulvan	17.9 ± 0.3%	[156]
Pressure Liquid Extraction (PLE) (103 bar 2 cycle 140 °C 15 min)	Yes	*F. virsoides C. barbata*	All polysaccharides content	*F. virsoides* 10.22 ± 0.03 *C. barbata 11.77 ±* 0.03	[150]
Microwave-Assisted Extraction (MAE) (80 °C 10 min)	Yes	*F. virsoides C. barbata*	All polysaccharides content	*F. virsoides* 13.19% *C. barbata* 6.43%	[150]
Hot Water Extraction (HWE) 90 °C 3 h)	Yes	*Ulva lactuca*	Ulvan	18.61%	[157]

**Table 4:** Functional activities of algal polysaccharides in food products.

Food Products	Algae/Polysaccharides	Optimal Concentration	Activity	References
Yogurt	Fucoidan from *Sargassum christaefolium*	0.2 g/100 mL	t has increased prebiotic and antibacterial activity.	[165]
Fresh cheese	*Fucus spiralis* and *Petalonia binghamiae* polysaccharides *	0.5 g/100 g	The weight loss of the cheeses was prevented, and the cheeses had a high antioxidant content.	[169]
Beef sausage	Ulvan from *Ulva rigida*	0.5 g/100 g	It has significantly reduced the cooking loss of sausage and increased its moisture content. Ulvan has provided antioxidant activity.	[168]
Meat and mince mixtures, sausage	Kappa-Carrageenan **	0.2 g/100 g	The addition of kappa-carrageenan has improved quality of products.	[170]
Functional cottage cheese	Alginate ** Carrageenan **	1 g/100 g alginate 1 g/100 g carrageenan	Under simulated gastrointestinal conditions, coating with alginate-carrageenan gums increased the viability of probiotics.	[166]
Dairy products	Alginate **	1.5 g/100 mL	It has achieved microencapsulation of the contents by embedding a component in a micron-sized protective matrix.	[171]
Buckwheat noodle	Alginate from brown algae	1 g/100 g	It reduced cooking loss, pGI and surface stickiness of noodles	[162]
Wheat starch and noodle	Laminarin from *Laminaria*	6 g/100 g	It has strongly inhibted the in vitro digestibility of starch. Also, it improves the cooking textural properties of noodles. It reduces cooking loss and stickiness increase water absorption and firmness.	[161]
Chicken sausage	Alginate **	4 g/100 g	It reduced the values indicating lipid oxidation and cooking loss. It also extended shelf life by providing antioxidant activity and microbial balance.	[163]
Fresh cheese	Alginate **	2 g/100 mL	Alginate-based edible coatings containing *Lactococcus* strains that produce bacteriocins have been effective in reducing *Listeria monocytogenes* cells in fresh cheese.	[172]
Poultry meat	Alginate, fucose and other sulfated polysaccharides from *Laminaria digitata*	15% *l. digitata* addition to the diet	The addition of 15% *L. digitata* to the diet increased the nutritional value and antioxidant pigments of poultry meat.	[173]
Guava fruits coating	Alginate **	2 g/100 mL	Alginate coating has been added to black cumin extract and has delayed guava’s respiration rate, weight loss, hardness loss, color change, and carotenoid formation. In addition, the fruit has increased vitamin C, total phenolic and flavonoid content, and antioxidant, antidiabetic activity.	[174]
Bakery products	Kappa-Carrageenan from *Eucheuma*	10 g/100 g	The addition of kappa-carrageenan to the starch-gluten system increased viscosity and provided a more stable and firm structure.	[175]
Meat	Pea protein with Agar **	1.5 g/100 g	It has been integrated into meat emulsions, replacing the physical location of fat, thereby reducing the fat and energy content of the product while increasing emulsion stability, cooking efficiency, and nutritional value.	[176]
Curd cheese	Agar **	n.d.	The agar-based active film significantly reduced the microbial load by reducing thermotolerant coliforms and coagulase-positive staphylococci.	[177]
Bread	Fucoidan from *Undaria pinnatifida*	0.4 g/100 g	Specific bread volume increase, softer texture	[160]
Yogurt	Ulvan **	1 g/100 mL and 2 g/100 mL show similar activities	*Lactobacillus acidophilus* (LA-5) *Streptococcus thermophilus* (TH-4) Bifidobacterium sp. increase The consistency, viscosity, and hardness of the yogurt increase.	[178]
Bovine meat	Alginate from brown alga species **	1 g/100 ml	Alginate coatings have been used for active food packaging applications. When used with pineapple peel, it exhibits antimicrobial and antioxidant effects.	[179]
Whey	*Ulva* biomass *	50–75 g/100 g	Biomethane has been produced through waste management.	[180]

* A Specific type of polysaccharide is not mentioned. ** A specific type of algae is not mentioned.

## Data Availability

No new data were created or analyzed in this study. Data sharing is not applicable to this article.
